# The Effect of Age and Intrinsic Aerobic Exercise Capacity on the Expression of Inflammation and Remodeling Markers in Rat Achilles Tendons

**DOI:** 10.3390/ijms23010079

**Published:** 2021-12-22

**Authors:** Runa Kinitz, Estelle Heyne, Lauren G. Koch, Steven L. Britton, Manuela Thierbach, Britt Wildemann

**Affiliations:** 1 Experimental Trauma Surgery, Department of Trauma, Hand and Reconstructive Surgery, Jena University Hospital, Friedrich Schiller University Jena, 07747 Jena, Germany; runa.kinitz@uni-jena.de (R.K.); manuela.thierbach@med.uni-jena.de (M.T.); 2 Department of Cardiothoracic Surgery, Jena University Hospital, 07747 Jena, Germany; Estelle.heyne@med.uni-jena.de; 3 Department of Physiology and Pharmacology, University of Toledo, Toledo, OH 43606, USA; Lauren.Koch2@UToledo.edu; 4 Department of Molecular and Integrative Physiology, University of Michigan, Ann Arbor, MI 48109-5048, USA; brittons@med.umich.edu

**Keywords:** tendinopathy, inflammation, aging, tendon histology, rat

## Abstract

Old age, adiposity, and metabolic disorders are known as risk factors for chronic tendinopathy, which is a common problem in both athletes and the general population. However, the importance of these influencing factors has not yet been well understood. This study investigated alterations in gene expression and histology of Achilles tendons of young (10 weeks) and old (100 weeks) rats bred for low (low capacity runners, LCR) and high (high capacity runners, HCR) intrinsic aerobic exercise capacity. In this rat model, LCR displayed a phenotype of reduced exercise capacity, higher body weight, and metabolic dysfunctions compared to HCR. We hypothesized that the risk factors for tendinopathy in old LCR could lead to more pronounced impairments in Achilles tendon tissue. In quantitative real-time PCR (qPCR), age-related downregulation of tenocyte markers e.g., tenomodulin, genes related to matrix modeling and remodeling (e.g., collagens, elastin, biglycan, fibronectin, tenascin C) as well as transforming growth factor beta 3 (*Tgfb3*) have been detected. Inflammation marker cyclooxygenase 2 (*Cox2*) was downregulated in old rats, while microsomal prostaglandin E synthase 2 (*Ptges2*) was upregulated in old HCR and old LCR. In all groups, interleukin 6 (*Il6*), interleukin 1 beta (*Il1b*), and tumor necrosis factor alpha (*Tnfa*) showed no significant alteration. In histological evaluation, tendons of old rats had fewer and more elongated tenocyte nuclei than young rats. Even though a higher content of glycosaminoglycans, a sign of degeneration, was found in old HCR and LCR, no further signs of tendinopathy were detectable in tendons of old rats by histological evaluation. Low intrinsic aerobic exercise capacity and the associated phenotype did not show significant effects on gene expression and tendon histology. These findings indicate that aging seems to play a prominent role in molecular and structural alterations of Achilles tendon tissue and suggests that other risk factors associated with intrinsic aerobic exercise capacity are less influential in this rat model.

## 1. Introduction

Achilles tendinopathy is a common disease among athletes, especially runners [1]. Nevertheless, it also occurs among non-athletes [2] and is present in aged patients [3]. Tendinopathy is a clinical diagnosis characterized by a combination of pain, swelling, and impaired performance [4] in which overuse is one of the main risk factors for the occurrence in the Achilles tendon [5,6,7]. Further risk factors for the development of tendinopathy include adiposity and insulin resistance [3,8,9,10,11], but the pathogenesis remains unclear. Tendinopathy is currently understood as a degeneration process in which inflammation, especially in the early onset of tendinopathy, is widely discussed [6,12,13,14,15]. Tenocytes, the tendon’s fibroblast-like stromal cells, constitute 90–95% of the cells in tendons [16] and can be characterized by the expression of scleraxis *(Scx),* mohawk *(Mkx),* tenomodulin (*Tnmd*), and early growth response 1 *(Egr1)* [17]. Because *Scx, Mkx*, and *Tnmd* were found to be downregulated in aged flexor digitorum longus tendons of mice, alteration in tenocyte markers might promote tendon degeneration [18]. Furthermore, *Tnmd* seems to be important in early tendon healing as *Tnmd* knockout (*Tnmd*^-/-^) mice showed reduced cell proliferation and downregulation of tendon-related genes in an Achilles tendon injury model published by Lin et al. [19]. Tenocytes are involved in modeling processes of tendon tissue by producing collagens and releasing proteases [16]. Collagens are an essential part of the extracellular matrix (ECM) of tendons [12]. While collagen type 1 (*COL1A1*), collagen type 3 (*COL3A1*), and collagen type 5 (*COL5A2*) are often found to be altered in tendinopathy and chronic ruptures of human Achilles tendons [20,21], aging seems to be associated with the downregulation of collagens in mice and rats [18,22]. Matrix metalloproteinases (MMPs) are involved in tendon remodeling processes and can both promote tendon healing and ECM breakdown [23]. *MMP1* is upregulated in human tendinopathic patellar tendons [24], while *MMP3* is downregulated in human Achilles tendon disorder [21,25]. Inflammatory mediators seem to trigger matrix degradation as interleukin 1 beta (IL1b) induces the expression of matrix degrading enzymes such as *MMP1*, *MMP3*, and other inflammatory markers such as *COX2* and prostaglandin E2 (PGE2) synthesis in human tendon cells [26,27,28]. Upon analysis of injured superficial digital flexors in horse tendons, a positive correlation of age and PGE2 level can be found, while the correlation is negative in uninjured tendons. These findings suggest a so called inflamm-aging process in injured tendons [29]. Additionally, injections of prostaglandin E2 in Achilles tendons of rats and patellar tendons of rabbits cause tendon degeneration on a histological level [30,31].

On the histological level, tendinopathy is characterized by a loss of collagen fiber structure, hypercellularity, rounding of tenocyte nuclei, hyalinization, fat infiltration, hypervascularity, and increased glycosaminoglycan (GAG) content [5,20,32]. The presence of inflammatory cells such as neutrophils and macrophages in tendinopathic tissue is still under debate [12,15]. Scott et al. found histological changes in rat tendons after excessive exercise, but inflammatory cells were not present [5]. An immunohistochemical analysis of human Achilles tendon tissue revealed an increase in CD45+ cells in acute and chronic ruptures when compared with tendinopathic tendons [20].

Taking all this into account, tendinopathy can be described as a disease with multiple influencing factors in which the contribution of individual risk factors to the onset of disease is still not completely understood. Therefore, this study aimed to characterize the effect of age and metabolic dysfunctions on female rat Achilles tendons. Achilles tendons of young (10 weeks) and old (100 weeks) rats bred for low (low capacity runners, LCR) and high (high capacity runners, HCR) intrinsic aerobic exercise capacity were studied [33]. In this rat model, low intrinsic aerobic exercise capacity is associated with features of the metabolic syndrome including more visceral adiposity, higher plasma triglycerides, and elevated plasma free fatty acids [34,35]. We hypothesize that old age and low intrinsic aerobic exercise capacity lead to molecular alterations in matrix production, remodeling, and inflammation of tendon tissue. Furthermore, we hypothesized that old age and low intrinsic aerobic exercise capacity and the associated phenotype affect tendons on a structural level.

## 2. Results

Four groups (HCR 10 weeks, LCR 10 weeks, HCR 100 weeks, LCR 100 weeks) of female rats (n = 7 per group) bred for intrinsic high (HCR) or low aerobic running capacity (LCR) were studied. As described earlier, these rat groups differed significantly for inherited maximal running distance, and low intrinsic running capacity led to metabolic dysfunctions and lower life span [33,35,36,37]. The body weight of old LCR rats was significantly higher comparted to old HCR rats. Old rats of both breeds had a higher tendon weight compared to young rats, whereas RNA quantity as an approximation for cell number was significantly lower in old rats compared to young rats (Table 1).

### 2.1. Gene Expression

The gene expression of markers for tenocytes (*Tnmd, Egr1, Scx, Mkx*), markers for modeling and remodeling of ECM (*Col1a1, Col1a3, Bgn, Dcn, Eln, Fbn1, Tnc, Fn1, Postn, Mmp1, Mmp3, Mmp9*), and growth factors (*Tgfb1, -3*) was analyzed to detect alterations in tendon metabolism. The gene expression of inflammatory (*Cox2, Ptges2, Ptger4, Il1a, Il1b, Il6, Il33, Tnfa, Hmgb1*) and neuroinflammatory (*Tac1, Tacr1*) markers was analyzed to assess the role of inflammation in tendon aging and tendon pathologies. This investigation was performed to obtain an overall impression of molecular alterations. In total, 16 of the 29 genes investigated in the qRT-PCR showed significant differences. The strongest age-related effects were detected in markers for modeling and remodeling of ECM.

#### 2.1.1. Tenocyte Markers

**Figure 1:**
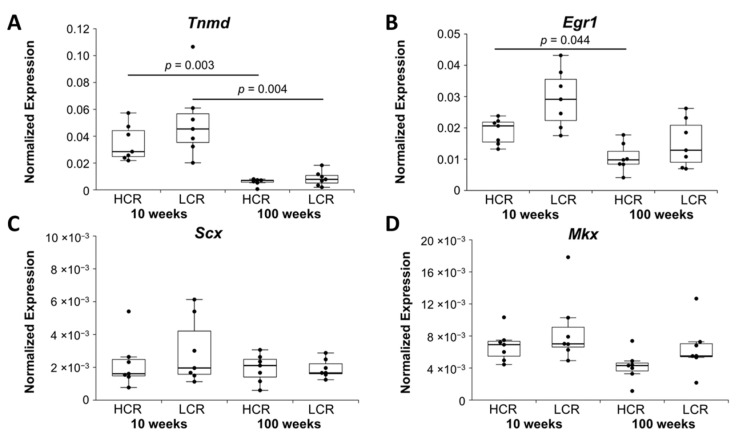
Normalized expression (NE) of tenocyte markers (**A**) tenomodulin, (**B**) early growth response 1, (**C**) scleraxis, and (**D**) mohawk. qRT-PCR data were normalized to the expression of the house keeping gene *Rps18*. Results are shown as box plots with individual data points. Significant differences between two groups are marked as a line spanning between them with the *p*-value displayed above. *p ≤* 0.05 was considered statistically significant.

The tendon related proteoglycan tenomodulin *(Tnmd)* was downregulated in old HCR and old LCR compared to young HCR and young LCR, respectively (Figure 1A). The zinc finger transcription factor early growth response 1 *(Egr1)* was also downregulated in the old HCR and old LCR (Figure 1B), but downregulation in old LCR compared to young LCR just missed significance after Bonferroni–Holm correction (*p =* 0.051). The tendon related transcription factors scleraxis (*Scx*) and mohawk (*Mkx*) did not show significant differences (Figure 1C,D).

#### 2.1.2. Markers of Modeling and Remodeling of ECM

**Figure 2:**
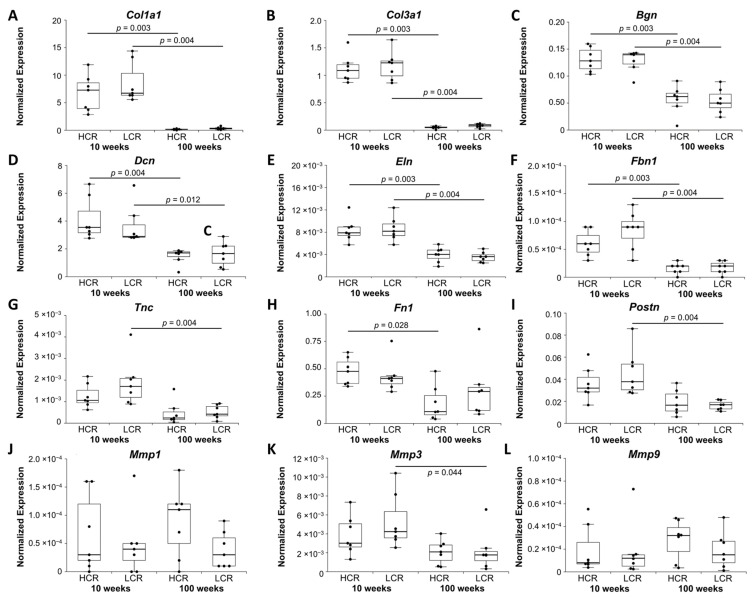
Normalized expression (NE) of tendon-associated (**A**,**B**) collagens, (**C**–**I**) other ECM modeling and remodeling genes, and (**J**–**L**) MMPs. qRT-PCR data were normalized to the expression of the house keeping gene *Rps18*. Results are shown as box plots with individual data points. Significant differences between two groups are marked as a line spanning between them with the *p*-value displayed above. *p ≤* 0.05 was considered statistically significant.

*Col1a1* and *Col3a1* were downregulated in old HCR and old LCR compared to the respective young ones (Figure 2A,B). *Col1/Col3* expression ratio was lower in old HCR compared to young HCR, indicating that old HCR expressed more *Col3a1* in relation to *Col1a1*. However, alteration in the *Col1/Col3* expression ratio did not remain significant after the Bonferroni–Holm correction (*p* = 0.128, data not shown). Additionally, biglycan (*Bgn*), decorin (*Dcn*)*,* elastin (*Eln*), and fibrillin 1 (*Fbn1*)*,* as components of ECM were all downregulated in old HCR and old LCR (Figure 2C–F). Fibronectin 1 *(Fn1)* was only downregulated in old HCR compared to young HCR (Figure 2) while periostin *(Postn)* and tenascin C *(Tnc)* were only downregulated in old LCR compared to young LCR (Figure 2G,I).

To gain further information about matrix degeneration, the expression of matrix degrading enzymes was investigated. The collagenase matrix metalloproteinase 3 *(Mmp3)* was downregulated in old LCR compared to young LCR. The collagenase *Mmp1* and gelatinase *Mmp9* showed no statistical differences (Figure 2J–L).

#### 2.1.3. Growth Factors

**Figure 3:**
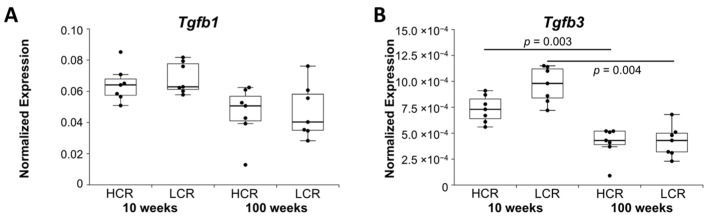
Normalized expression (NE) of (**A**) transforming growth factor beta 1 and (**B**) beta 3. qRT-PCR data were normalized to the expression of the house keeping gene *Rps18*. Results are shown as box plots with individual data points. Significant differences between two groups are marked as a line spanning between them with the *p*-value displayed above. *p ≤* 0.05 was considered statistically significant.

The expression of transforming growth factor beta 1 and 3 *(Tgfb1, -3)* was investigated. Expression of *Tgfb1* was downregulated in old HCR and old LCR compared to young HCR and young LCR, respectively, but did not remain significant after the Bonferroni–Holm correction (*p =* 0.078, *p =* 0.104) (Figure 3A). *Tgfb3* showed significant downregulation in old HCR and old LCR (Figure 3B).

#### 2.1.4. Inflammatory and Neuroinflammatory Markers

**Figure 4:**
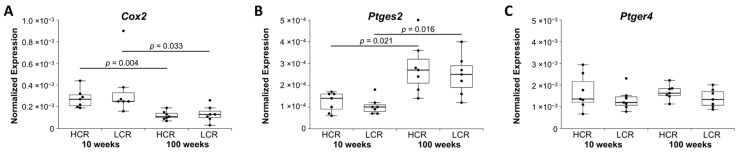
Normalized expression (NE) of (**A**,**B**) synthases and (**C**) receptor of prostaglandin E2. qRT-PCR data were normalized to the expression of the house keeping gene *Rps18*. Results are shown as box plots with individual data points. Significant differences between two groups are marked as a line spanning between them with the *p*-value displayed above. *p ≤* 0.05 was considered statistically significant.

Cyclooxygenase 2 (*Cox2*) and microsomal prostaglandin E synthase 2 (*Ptges2*) are involved in the synthesis of PGE2. *Cox2* was downregulated in old HCR and old LCR compared to young HCR and young LCR, respectively (Figure 4A). In contrast, *Ptges2* was upregulated in old HCR and old LCR compared to young HCR and young LCR, respectively (Figure 4B). However, prostaglandin E2 receptor EP4 subtype *(Ptger4)* showed no change in expression (Figure 4C).

**Figure 5:**
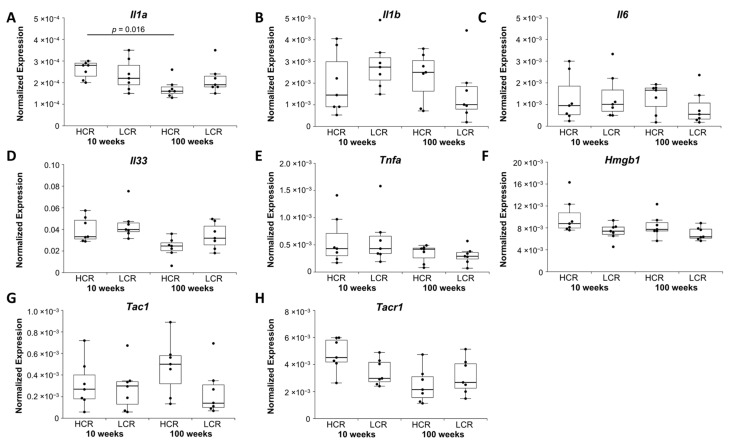
Normalized expression (NE) of (**A**–**D**) interleukins Il6, Il1b, Il33, Il1a, (**E**) tumor necrosis factor alpha, (**F**) high mobility group protein box 1 protein *(Hmgb1)*, and (**G**) synthase and (**H**) receptor of substance P. qRT-PCR data were normalized to the expression of the house keeping gene *Rps18*. Results are shown as box plots with individual data points. Significant differences between two groups are marked as a line spanning between them with the *p*-value displayed above. *p ≤* 0.05 was considered statistically significant.

Interleukin 1 alpha *(Il1a)* was downregulated in old HCR compared to young HCR (Figure 5A). Interleukin 1 beta *(Il1b)* and interleukin 6 *(Il6)* showed no change in expression (Figure 5B,C). Interleukin 33 *(Il33)* was downregulated in old HCR compared to young HCR, but the differences did not remain significant after Bonferroni–Holm correction (*p =* 0.068, Figure 5D). Tumor necrosis factor alpha *(Tnfa)* did not show any change in expression (Figure 5E). Expression of high mobility group protein box 1 protein *(Hmgb1),* which is associated with necrosis and senescence, was also not altered (Figure 5F). Protachykinin 1 (*Tac1*) transcribes for substance P and neurokinin A and is a marker for neuroinflammation. Substance P modulates tissue remodeling, vascular flow, and pain in tendons. While *Tac1* showed no change in expression (Figure 5G), downregulation of tachykinin receptor 1 *(Tacr1)* in old HCR compared to young HCR just missed significance after Bonferroni–Holm correction (*p =* 0.068, Figure 5H).

#### 2.1.5. Summary of Gene Expression Results

Taking all gene expression results into account, age-related alterations were observed in most of the markers of modeling and remodeling of ECM. Most investigated genes showed a downregulation in old rats (Table 2, highlighted in blue), while only *Ptges2* showed an upregulation in old rats (Table 2, highlighted in red). Gene alterations due to intrinsic aerobic exercise capacity were not detected.

### 2.2. Histology of the Achilles Tendon

**Figure 6:**
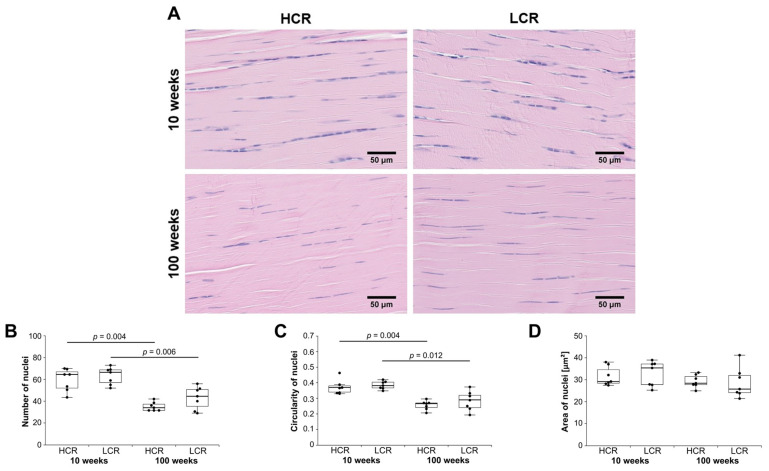
Evaluation of tenocyte nuclei. (**A**) Exemplary images of mid-portion Achilles tendon of young and old HCR and young and old LCR stained with hematoxylin and eosin (HE). Old HCR and old LCR showed a lower cellularity and more elongated tenocyte nuclei. Scale bar: 50 μm. (**B**) Number of tenocyte nuclei, (**C**) circularity of tenocyte nuclei in which a value of 1 indicates a perfect circle and a value of 0 indicates a very elongated polygon, and (**D**) area of tenocyte nuclei in μm^2^. Results are shown as box plots with individual data points. Significant differences between two groups are marked as a line spanning between them with the *p*-value displayed above. *p ≤* 0.05 was considered statistically significant.

Histological analyses were performed to investigate possible cellular and structural differences of the Achilles tendons between the groups. As tendons consist of myotendinous junction (MTJ), mid-portion, and enthesis, the morphology and cell density of tenocytes is not homogenous. Because tendinopathy is often located in the mid-portion of the Achilles tendon, analysis of tenocyte nuclei was performed in this area. Number of nuclei, circularity of nuclei, and area of nuclei was evaluated (Figure 6A). Morphological differences of tenocyte nuclei between groups were found by performing semi-automated image analysis. The number of nuclei was significantly lower in the old HCR and old LCR compared to the young HCR and young LCR, respectively (Figure 6B). The circularity of the nuclei was lower in the old HCR and old LCR compared to the young HCR and young LCR, respectively, meaning that the tenocyte nuclei of the young rats were rounder in shape (Figure 6C). The area of nuclei, defined as area of all counted nuclei in μm^2^, was not significantly altered between groups (Figure 6D). No significant differences due to intrinsic aerobic exercise capacity could be found.

#### Histological Score

**Figure 7:**
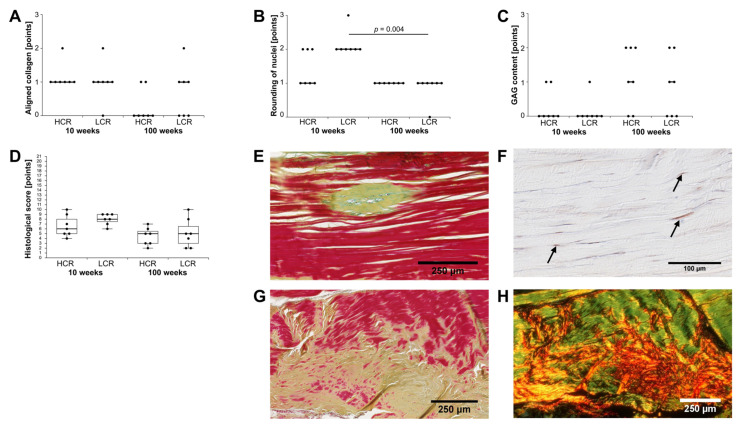
Semi-quantitative evaluation of (**A**) alignment of collagen, (**B**) rounding of tenocyte nuclei, and (**C**) GAG-content. Data are given as individual dot plots with median as a line in between the data points. (**D**) Total modified Movin score is given as a box plot with individual data points. Significant differences between two groups are marked as a line spanning between them with the *p*-value displayed above. *p ≤* 0.05 was considered statistically significant. Exemplary images of histological evaluation show an (**E**) abnormal amount of the appearance of GAG in old rats (HCR 100 weeks, stained yellow-green in Movat Pentachrome (MP) staining, scale bar: 250 μm) and (**F**) αSMA positive myofibroblasts (arrows) in young rats (HCR 10 weeks, stained red in αSMA staining, scale bar: 100 μm). Disorientated collagen in the mid portion of the same tendon was also documented (LCR 10 weeks), stained in (**G**) Movat Pentachrome, scale bar: 250 μm and (**H**) Picro Sirius Red with polarization microscopy, scale bar: 250 μm.

A modified Movin score (0 = normal tissue, higher value = altered tissue, for details see Section 4) was performed to evaluate the characteristics of tendinopathy over the entire Achilles tendon. Old HCR showed a slightly higher amount of aligned collagen, which means a lower histological scoring (Figure 7A). This difference just missed significance after Bonferroni–Holm correction (*p =* 0.064). Tendon architecture was not significantly different between groups (data not shown). Exemplary images of different stainings showed tendon areas where collagen bundles lost parallel arrangement (Figure 7G,H). Disorientation of collagen bundles might influence tendon functionality. Young LCR also showed significantly more rounded tenocyte nuclei compared to old LCR (Figure 7B). When evaluating the glycosaminoglycan (GAG)-content, higher GAG-content (classified as 2 or 3) was only found in old HCR and old LCR (Figure 7C,E). Accumulation of GAG between collagen fibers might impair fiber cohesion and lead to reduced elasticity. Differences in the amount of fat within the tendon tissue and vascularization were not present (data not shown). Taken together, the histopathological score was slightly higher in young rats, especially young LCR compared to old LCR, but without statistical significance (*p* = 0.24, Figure 7D). When evaluating the vascularization, alpha smooth muscle actin (αSMA) positive cells (so-called myofibroblasts) were observed in some tendons. Myofibroblasts were present in four out of seven young HCR and four out of seven young LCR, while myofibroblasts were not found in old HCR and only in one out of seven in old LCR (Figure 7F).

## 3. Discussion

Health impairments due to tendinopathies are a common problem in sports. The Achilles tendon, in particular, is often affected in endurance runners [1]. The fact that 95 out of 350 athletes of both sexes were diagnosed with Achilles tendinopathy at the 2017 Marathon of Rome is a striking example of this [38]. Even though overuse is one of the main risk factors for Achilles tendinopathy, it is a multifactorial disease that also occurs in non-athletes [2,9]. Risk factors are divided into intrinsic and extrinsic factors [9]. The incidence of Achilles tendinopathy increases in older people [3,38], showing that aging belongs to the intrinsic risk factors [39]. Furthermore, metabolic disorders such as insulin resistance, hyperlipidemia, and diabetes are associated with Achilles tendinopathy and are known as intrinsic risk factors [9,11,40,41]. A recent study investigating the effect of running on weight reduction showed that regular runners with mid-portion Achilles tendinopathy had a worse metabolic profile than uninjured regular runners [40].

The current study is an attempt to understand the contribution of age and low intrinsic aerobic exercise capacity as intrinsic risk factors to the onset of Achilles tendinopathy. Until now, low intrinsic aerobic exercise capacity is not known to be a risk factor for tendinopathy. The chosen animal model, however, is also associated with a wide range of metabolic dysfunctions that can be summarized as a metabolic syndrome, which is associated with tendinopathy [36]. Given that both age and metabolic disorders are responsible for the onset of tendinopathy, the aim of the present study was to evaluate whether the former or the latter would have the greater effect on Achilles tendons or if additive effects would occur in old LCR. Therefore, the rat model of breeding according to intrinsic aerobic exercise capacity is a useful model to determine how metabolic dysfunction influences tendon tissue in rats of 10 weeks and 100 weeks of age.

Significant age-related changes in gene expression were seen in this study. Collagens *Col1a1* and *Col3a1* were downregulated in old HCR and old LCR compared to young rats, while differences in the *Col1a1/Col3a1* expression ratio showed the tendency to decrease in old HCR and old LCR without statistical significance. *Bgn, Dcn, Eln*, and *Fbn1* as components of ECM were also downregulated in old HCR and old LCR. Downregulation of collagens due to age is consistent with previous findings in the flexor tendons of mice [18] and in the Achilles and tibialis anterior tendons of rats [22]. However, Yu. et al. found no regulation of *Col1a1* in the Achilles tenocytes of 24-month-old rats [42]. The decreased expression of collagens and other components of tendon ECM in old HCR and old LCR might be a sign of lower modeling and remodeling activities in aged tendon tissue.

Even though age was shown as a risk factor for lower-extremity tendinopathies and prevalence is higher in aged human individuals [3], decreased expression of collagens seems not to be a common feature. In contrast, human affected Achilles tendons previously showed increased expression of collagens [20,21,43] without differences in the *Col1a1/Col3a1* expression ratio [20]. Elastin, biglycan, and decorin were previously found to be decreased with age in normal tendons of rats and humans [22,44]. *BGN* and *TNC* were upregulated in tendinopathy, while *DCN* showed no change [21,44]. Although regulation of ECM markers is opposite in aging and tendinopathy, decreased expression of ECM components in age might lead to a lower ability of matrix formation and increased risk of injury [22].

Alterations in expression of matrix metalloproteinases (Mmps) were demonstrated in tendinopathy [23]. In this study, expression of *Mmp1*, *Mmp3*, and *Mmp9* was investigated. Only *Mmp3* showed a significant change and was significantly downregulated in old LCR compared to young LCR. Decreased expression of *Mmp3* with age is consistent with previous findings in human Achilles tendinopathy and may indicate that it is necessary to prevent tendinopathic changes [21,25]. In contrast, *MMP1* was upregulated in human patellar tendinopathy [24] and increased expression of *MMP1* was found in tendon ruptures [20,45,46] Additionally, upregulation of *MMP2* and *MMP9* was shown to be a feature of tendinopathy [20,43] and increased MMP9 activity was seen after exercise [47,48]. Increased expression of *Mmp2* and *Mmp9* was not only seen in tendinopathy, but also during aging. In Achilles tendons of aged rats, *Mmp2* and *Mmp9* were observed at an increased level of expression and enzymatic activity [42]. However, alteration of *Mmps* was not very pronounced in this study and might indicate that pathological tendon remodeling is not a central feature of this rat model.

In the group of tenocyte markers, diminished expression of tenomodulin *(Tnmd)* was present in old HCR and old LCR while early growth response 1 *(Egr1)* was only downregulated in old HCR. Downregulation of *Tnmd* due to age is consistent with previous findings, while downregulation of *Scx* and *Mkx* such as that found in flexor digitorum longus tendons of mice was not confirmed [18]. *Tnmd* had an important role in self-renewal and reducing senescence, while reduction in *Tnmd* expression impaired tendon healing [19,49]. Furthermore, *TNMD* was important for the functional performance of tendons [50]. Therefore, downregulated expression of *Tnmd* in old HCR and old LCR might result in alterations in tendon functionality. This suggestion may also apply to *Egr1*, as tendons from *Egr1* knockout (*Egr1*^−/−^) mice showed a decreased expression of collagens and a mechanical weakness [51].

In the past years, the contribution of inflammatory processes to the development of tendinopathies has been frequently discussed [12,13,14,15], and overlapping features of mechanical stress related tendon disorders and chronic inflammatory arthritis have been described [17]. Nuclear factor kappa B (NF-κB) was identified as a possible target in chronic tendon disease [52,53]. This transcription factor induced inflammation by regulating the expression of cytokines and adhesion molecules [54]. In the present study, the gene expression levels of several inflammatory markers were investigated. The expression of *Cox2* was decreased in old HCR and old LCR. In the case of *Cox2*, findings in the literature are ambiguous. In human patellar tendinopathy, immunohistochemical analysis indicated an elevated expression of *COX2* [55]. In human Achilles tendinopathy, either upregulation of *COX2* or no regulation was observed [20,28,56]. In injured equine superficial digital flexor tendons, functionally equivalent to human Achilles tendons, no *Cox2* changes due to injury or age were detected [29]. Interestingly, in this study, microsomal prostaglandin E synthase 2 *(Ptges2)*, encoding for an enzyme that converts (Cox)-derived prostaglandin H2 (PGH2) to prostaglandin E2 (PGE2) [57], was the only significantly upregulated gene in old HCR and old LCR. This finding is consistent with previous investigations of both tendinopathic and aged tendons and in response to repetitive mechanical loading [28,29,58]. Dakin et al. hypothesized that upregulated *Ptges2* in aged tendons contributes to the process of so called inflamm-aging [29]. Injections of Il1b also induced arachidonic acid metabolism and stimulated the expression of prostaglandin E2 receptor EP4 subtype [26,27]. However, neither *Il1b* nor *Ptger4* showed an altered expression, which is consistent with previous findings [20,29]. As such, *Ptges2* stands out as the only upregulated gene in old HCR and LCR and makes it an interesting target for further analysis of its role in tendon aging.

In addition to inflammatory markers, neuroinflammation was investigated in the present study, as substance P and its receptor tachykinin receptor 1 are involved in tenocyte proliferation, angiogenesis, and pain, all of which are features of tendinopathy. While pain as a feature of tendinopathy was not addressed in this study, no signs of pathological tenocyte proliferation or hypervascularization were seen. The lack of histological consequences of upregulated expression of substance P and its receptor fits the unaltered expression of tachykinin receptor 1 *(Tacr1)* and tachykinin 1 gene *(Tac1),* encoding for substance P and neurokinin A. Nevertheless, substance P was also discussed as a component of tendon healing [59,60,61,62,63,64].

Another marker that promotes tenocyte proliferation is transforming growth factor beta (*Tgfb1, -2, -3*) [65]. Both increased and unaltered expression of *Tgfb1* in aged and tendinopathic tendons were described in previous studies [20,22,43,55]. *TGFB1* showed an unsignificant upregulation in previous early human patellar tendinopathy studies [66]. In this study, *Tgfb3* was significantly downregulated in old HCR and old LCR. The TGFb-superfamily is involved in tissue fibrosis and therefore the downregulation of TGFb pathways in tendon disease has been proposed to be a protective response to limit disease-associated fibrosis [67]. Therefore, the decreased expression of *Tgfb3* in old HCR and old LCR in this study might counteract the reduced ability of tissue modeling and remodeling in aged rats.

Looking at the histological results of this study, a decrease in the number of tenocytes and a more elongated shape of tenocyte nuclei, as a typical feature for tenocytes while tenoblasts can also be ovoid [16], were found in old HCR and old LCR tendons. These findings were consistent with previous studies on mice and sheep [18,68]. Loss in cell density with age might lead to a decrease in metabolic activity and impaired remodeling and healing [69]. However, the histological appearance of tendinopathy was previously characterized by loss of collagen fiber structure, hypercellularity, rounding of tenocyte nuclei, hyalinization, fat infiltration hypervascularity, and increased glycosaminoglycan (GAG) content [32]. Even though aging and tendinopathy appeared differently in histology, an increase in GAG-content might be a common feature, since high amounts of GAG were observed only in old HCR and old LCR. Regarding the changes in cell density, it was previously assumed that decrease in cellularity is more likely to be a feature of maturation rather than aging [68,70]. This might also explain why the modified Movin score of Achilles tendons showed no significant differences and even a tendency of higher scoring in young HCR and young LCR in the present study was observed. At this point, it is important to mention that the original Movin score was not used for age-group comparisons and therefore might be limited in evaluating tendon pathologies in different age groups [32]. αSMA-positive myofibroblasts were detected in immature tendons [68] and in this study were more often detected in young HCR and LCR. This indicates that myofibroblasts were not only present after exercise and in tendon healing [71], but also in tendon maturation.

Picro Sirius Red staining with polarization microscopy was previously described as a method to evaluate collagen content and orientation of collagen fibers [72,73,74]. Even the differentiation of collagen types has been claimed and discussed controversially [75,76]. For the present study, Picro Sirius Red staining was taken into consideration for collagen analysis. In our analysis, changes in color relating to stage rotation and possibly relating to collagen type were observed and in agreement with earlier findings [76]. Disorientated collagen was clearly visible in Picro Sirius Red staining, but did not provide any added value to Movat Pentachrome staining.

### Limitations

Several analyzed markers interact with each other and are part of signaling cascades such as interleukins and TNFa, which stimulate MMPs and PGE2 synthesis and cause ECM breakdown [14]. This kind of interacting mechanism was outside the scope of the present study and molecular results are limited to a descriptive level. To validate qPCR data, quantitative protein analysis is a useful investigation. Because tendon material was limited, we elected instead to perform histological analysis to gain more information on the tendon’s morphology.

Biomechanical differences in the musculoskeletal system of rats and humans lead to limitations in the comparability of Achilles tendons between the species. As rodents are quadrupeds, mechanical load might affect their Achilles tendons differently when compared to humans. Additionally, metabolic dysfunctions might have different effects on the Achilles tendon. This could explain why no effects due to intrinsic aerobic exercise capacity were found in this study. In a previous study, uphill running did not lead to exacerbation of collagenase-induced pathological changes in the Achilles tendon of HCR. A possible cause of this would be the preference of HCR rats to run, but no comparison to LCR was made in that study [77].

Regarding the rat age groups of this study, using relatively young rats could have skewed the results. Even though 10-week-old rats have reached sexual maturity [78], determining musculoskeletal maturity is difficult in rats [79]. However, descriptive histological analysis of 10-week-old rats might be limited by the fact that the musculoskeletal system has not fully matured, as sexual maturity does not necessarily correlate with tendon maturation [68].

As tendinopathy is a multifactorial disease and mechanical overload is one of the main risk factors in humans, exposure of rats to treadmill running might have led to changes that are more pathological.

## 4. Materials and Methods

### 4.1. Animals

The rat model of this study was developed and previously described by L.G. Koch and S.L. Britton et al. [33]. Rats were constantly selectively bred for aerobic capacity. Artificial selection started with a population of genetically heterogenous N:NIH rats. Rats differed significantly in their intrinsic aerobic exercise capacity and were divided into high capacity runners (HCR) and low capacity runners (LCR). For this study, Achilles tendons of young (10 weeks) and old (100 weeks) LCR and HCR were investigated (young: offspring of mated rats from 41th generation; old: rats from 39th generation). Collected data from breeding confirmed previously published results on the phenotype of HCR and LCR [34,35,36,37] and body weights are shown in Table 1 (partly used from another rat cohort; young: offspring of mated rats from 33th). Rats were fed ad libitum and kept at 21 °C with a light cycle of 12 h. The use of animals was consistent with the Guide for the Care and Use of Laboratory Animals, published by the National Institutes of Health (NIH Publication no. 85-23, revised 1996). Achilles tendons from all rats were residual tissue from other investigations. These investigations followed experimental protocols, which were approved by the local authorities (Thüringer Landesamt für Verbraucherschutz, permission number: 02-082/14). By using residual tissue, we followed the 3R-principle to reduce animal experiments [80]. Animals were weighed and deep anesthesia was induced using thiopental (150 mg/kg bodyweight) before animals were sacrificed by cervical dislocation.

### 4.2. Sample Collection

Left and right Achilles tendons were explanted. The left Achilles tendon from each rat was used for gene expression analysis. The right Achilles tendon was used for histological evaluation and immunostaining. Achilles tendons were dissected from the muscular tendinous transition to the insertion at the calcaneus. Muscle and surrounding tissue were removed from the samples. For RNA-isolation, left Achilles tendons were frozen in liquid nitrogen and stored at −80 °C until RNA isolation was performed. For histological evaluation, right Achilles tendons were fixed in 4% paraformaldehyde at 4 °C for 24 h.

### 4.3. qPCR

Tendon samples were homogenized with 700 µL of TRIzol (Thermo Fisher Scientific, Waltham, MA, USA) using a T25 digital ULTRA-TURRAX (IKA, Staufen, Germany). Homogenized samples were incubated for 5 min at room temperature. For phase separation, 140 µL of chloroform was added, samples were shaken vigorously, and incubated for 10 min at room temperature. The samples were centrifuged at 12,000× *g* at 4 °C for 10 min. Then, the upper aqueous phase containing the RNA was transferred to a new tube, and the volume was measured. Samples were mixed with an equal volume of 70% ethanol. The RNA was further purified using the RNeasy Plus Mini Kit (Quiagen, Venlo, Netherlands) according to the manufacturer’s instructions. A NanoDrop 2000c spectrophotometer (Thermo Fisher Scientific, Waltham, MA, USA) was used to analyze the quantity and purity of the received RNA. RNA was stored at −80 °C until cDNA synthesis. cDNA synthesis was performed with 100 μg of RNA using qScript cDNA Supermix (Quanta Biosciences, Gaithersburg, MD, USA). qPCR was performed with SYBR ^®^ Green SuperMix (Quanta Biosciences) and a Rotor-Gene Q (Quiagen). For both, cDNA synthesis and qPCR, instructions in the manufacturer’s manual were followed. Sequences from GenBank (NCBI, Rockville Pike, MD, USA) and CLC Main Workbench 20 (Quiagen) software were used for primer design. All used primers are listed in Table 3. The primer pair for *Mmp9* was obtained from the literature [81], while the primer pair for *Scx* was obtained from Qiagen (QT01596028). All other primers were produced by Tib Molbiol, Berlin, Germany. Primers were tested for amplification efficiency in a dilution series on a cDNA pool. The cDNA pool contained cDNA of all samples. Primer specificity was validated in silico with BLAST (NCBI) and empirical with melting profiles. Rattus norvegicus ribosomal protein S18 *(Rps18)* was used as the reference gene. cDNA, primers, and SYBR^®^ Green SuperMix were stored at −20 °C. Three technical replicates were performed. All components were cooled on ice during preparation. No-template controls and positive controls were performed for each qPCR run. A cDNA pool of all samples with primers for the reference gene was used as the positive control. In the qPCR program, melting was performed at 95 °C for 5 min. Thermocycling was performed at 95 °C for 10 s, 57 °C for 15 s, and 72 °C for 20 s. Melting curve analyses were performed at 60 °C to 95 °C. The qPCR data were collected in Rotor Gene 6000 Series Software 1.7 (Qiagen). The threshold was set at 0.2 fluorescence units. After data collection, normalized expression (NE) was calculated [82] with the following formula:

(1) $$\mathrm{NE}=\frac{{\left({\mathrm{Primer}\;\mathrm{efficiency}}_{\mathrm{Reference}}\right)}^{C_{q}{}_{\mathrm{Reference}}}}{{\left({\mathrm{Primer}\;\mathrm{efficiency}}_{\mathrm{Target}}\right)}^{C_{q}{}_{\mathrm{Target}}}}$$

The implementation and reporting of the qPCR methods followed the MIQE Guidelines [83].

### 4.4. Histological Preparation

Samples were washed in phosphate-buffered saline for 1 h before automatic dehydration in a tissue processor. After dehydration, samples were embedded in paraffin. To prepare the samples for cutting, paraffin blocks were stored in the freezer at −20 °C for 24 h. Samples were cut in 5 µm sections with a rotary microtome (Leica RM1265, Nussloch, Germany). For further analyses, only sections from the central part of the tendons were chosen where the tendon was visible in its full extent. For histological staining, sections were deparaffinized and rehydrated in a descending alcohol series ending with ethanol 50%. Hematoxylin and eosin (HE) staining was performed with Mayer’s hemalum solution (Merck KGaA, Darmstadt, Germany) and eosin G 0.1% (Merck KGaA).

For Movat Pentachrome (MP) staining, rehydrated sections were pretreated with 3% acetic acid for 30 s before staining with 1% Alcian blue (Merck KGaA) for 30 min at room temperature. Then, sections were hydrated for 2 min and cell nuclei and elastic fibers were stained with Weigert’s Hematoxylin (Solution A and B 1:1, Waldeck GmbH & Co. KG, Münster, Germany). After hydration for 10 min, cytoplasm was stained with Brilliant-Crocein-Acid Fuchsine (Waldeck). Sections were differentiated with 1% acetic acid, pickled with 2% phosphortungstic acid (Carl Roth GmbH & Co. KG, Karlsruhe, Germany) for 15 min and again differentiated with 1% acetic acid. After dehydration with 100%, ethanol, collagen fibers were stained with Safron du Gatinais (Waldeck).

Picro Sirius Red staining was performed with a 1 h incubation with a staining kit (DIANOVA, Hamburg, Germany). Immunohistochemistry with a primary antibody against αSMA was also performed (see details below). Sections were digitized with a Nano Zoomer 2.0-HT (HAMAMATSU PHOTONICS Europe GmbH Herrsching, Germany) to perform automated image analysis and histopathological scoring.

### 4.5. Immunohistochemistry

Immunohistochemical staining was performed to analyze tendon vascularization. The tissue was blocked with 10% normal goat serum and 1% Triton X-100 and incubated with the primary antibody against αSMA (rabbit polyclonal to alpha smooth muscle actin, 1:200, Abcam, Cambridge, UK, ab5694) overnight at 4 °C. The staining was performed with the Vectastain ABC-HRP Kit (Vector Laboratories, Burlingame, CA, USA, PK-6101) and the Impact AEC Substrate Kit (Vector Laboratories, Burlingame, CA, USA, SK-4205) was used as a detection system. The slices were counterstained with Mayer’s hemalum solution (Merck, Darmstadt, Germany, 1:5) for 15 s. For the negative control, sections were stained without a primary antibody.

### 4.6. Semi-Automated Image Analysis

ImageJ 1.52p (Wayne Rasband, National Institute of Health, Bethesda, MD, USA) was used for automated image analysis. HE stained sections were used for this assessment. Two representative fields of 40× magnification from the mid-portion of each tendon were chosen. Fields had a size of 383 µm × 241 µm. These images were converted into 8-bit pictures and the threshold was adjusted manually to contrast tenocyte nuclei from surrounding tissue. Then, particle analysis was performed. The following parameters were assessed: number of nuclei, area of nuclei in µm^2^, and circularity of nuclei. The circularity was calculated with the following formula. A value of 1 in circularity indicates a perfect circle, while a value of 0 in circularity indicates a very elongated polygon.

(2) $$\mathrm{Circularity}=4\pi \;\times \;(\frac{\mathrm{area}}{\mathrm{perimeter}}{)}^{2}$$

### 4.7. Histological Score

A modified Movin score [32] was used to grade histological changes as described previously [45]. The following parameters were evaluated: tendon architecture, amount of aligned collagen, GAG content, fat content, cellularity, and vascularity. The parameter “rounding of nuclei” was taken from the original Movin score and added to the modification [32,45]. Each parameter was scored between 0 and 3, (0 = normal, 1 = slightly abnormal, 2 = abnormal, and 3 = markedly abnormal) with a maximum of 21 points (for classification, see [45]). Rounding of nuclei was classified from slender and spindle shaped (=0), slightly rounded (=1), rounded (=2) to strongly rounded (=3). The parameters were quantified visually by two independent observers in a blinded fashion. Observers quantified twice to determine intraobserver variability. In case of a discrepancy between the observers, results were discussed, and final score was determined together. Intraobserver and interobserver variability were measured by Kendall’s Tau c correlation (*p* < 0.01) analysis and revealed a strong association of 0.574 in intraobserver variability and a medium to strong association between 0.484 and 0.564 in interobserver variability.

### 4.8. Statistics

All statistical analyses were performed with SPSS 26 (IBM, Armonk, NY, USA). Nonparametric global Kruskal–Wallis test was used to determine significant differences between the four groups. Comparisons between the single groups were carried out with Mann–Whitney U tests. The Bonferroni–Holm correction was used to adjust the *p*-value. A *p*-value of ≤0.05 was considered statistically significant. Kendall’s Tau c correlation was used for measuring intra- and interobserver variability in the modified Movin score.

## 5. Conclusions

In summary, this study showed that diminished expression of genes related to ECM and tenocytes as well as reduced cellularity of Achilles tendons are significant features of tendon aging. These features indicate a reduced metabolic activity and predisposition to degenerative tendon diseases. Upregulation of *Ptges2* indicates the presence of inflammation in aged Achilles tendons, but inflammation does not seem to be a major feature in the investigated tendons. Age-related alterations are also present in Achilles tendon histology, but these changes are not to be assessed as pathological. Only the amount of GAG is a clear histological sign of degeneration in aged tendons. All in all, the present study points out aging as one predisposing factor for degenerative tendon diseases. Nevertheless, age-related changes in tendons might not automatically lead to the full extent of tendinopathy and clinical symptoms. It also emphasizes the multifactorial character of tendinopathy, because two risk factors, age and metabolic dysfunctions, were not enough to trigger the full picture of tendinopathy in this model. In the literature, clear evidence has been provided, showing that metabolic dysfunctions increase the risk of tendinopathy. Despite significant differences of HCR and LCR in metabolic parameters, metabolic dysfunctions played a minor role for tendon tissue in this rat model. To gain more knowledge about the meaning of the risk factors of tendinopathy, exposing different age groups of this rat model to mechanical overload is an interesting future direction.

## Figures and Tables

**Table 1:** Animal data.

	Young HCR	Old HCR	Young LCR	Old LCR
**Age in Weeks**	10.7 (10.2–10.9)	98.6 (97.8–99.1)	10.4 (10.1–10.6)	99.4 (97.8–99.9)
**Body weight [g]**	177.9 (168.3–185.0) ^#^	258.0 (244.5–291.8) ^†,##^	216.2 (196.1–230.6)	307.6 (198.0–323.5) ^‡^
**Achilles tendon weight [mg]**	18.0 (15.5–20.0)	30.0 (26.5–37.5) ^†^	18.0 (18.0–23.0)	32.0 (30.5–33.5) ^‡^
**RNA per tendon ration [μg/mg]**	0.34 (0.29–0.36)	0.14 (0.11–0.14) ^†^	0.40 (0.38–0.41)	0.11 (0.09–0.14) ^‡^

Data are presented as median and interquartile range (Q1-Q3). ^†^ Significant difference between old HCR and young HCR, ^‡^ Significant difference between old LCR and young LCR, ^#^ Significant difference between young HCR and young LCR, ^##^ Significant difference between old HCR and old LCR.

**Table 2:** Overview of investigated genes and significant alteration due to age.

Regulation	Function	HCR	LCR	No Changes
		100 vs. 10 Weeks	100 vs. 10 Weeks	
**Down**	Tenocyte markers	*Tnmd*		*Scx, Mkx*
		*Egr1*		
	ECM modelling & remodelling	*Col1a1, Col3a1, Bgn, Dcn, Eln, Fbn1*		*Mmp1, Mmp9*
		*Fn1*	*Mmp3, Postn, Tnc*	
	Growth factors	*Tgfb3*		*Tgfb1*
	Inflammation & Neuroinflammation marker	*Cox2*		*Il1b, Il6, Il33, Tnfa, Ptger4, Hmgb1, Tacr1, Tac1*
		*Il1a*		
**Up**	Inflammation marker	*Ptges2*		

Genes are grouped by down- and upregulation and by function. Overall, 15 genes showed downregulation in old HCR and/or LCR (highlighted blue). Genes that were downregulated in both old HCR and old LCR are displayed in the center of the column. Only *Ptges2* was upregulated in old HCR and LCR (highlighted red). Not differently regulated genes are shown in the rightmost column. *p* ≤ 0.05 was considered as statistically significant.

**Table 3:** qPCR primers.

Gene	Accession Number	Primer Sequence	Amplicon Size (bp)
*Bgn*	NM_017087.1	forward: 5′gattgagaatgggagcctga3′ reverse: 5′ccttggtgatgttgttggag3′	143
*Col1a1*	NM_053304.1	forward: 5′tgactggaagagcggagagt3′ reverse: 5′gatagcgacatcggcaggat3′	250
*Col3a1*	NM_032085.1	forward: 5′tgggatccaatgagggaga3′ reverse: 5′tcatggccttgcgtgttt3′	135
*Cox2*	U03389.1	forward: 5′agggagtctggaacattgtga3′ reverse: 5′attgtaagttggtgggctgt3′	107
*Dcn*	NM_024129.1	forward: 5′gcagggaatgaagggtctc3′ reverse: 5′tccacaacggtgatgctatt3′	195
*Egr1*	NM_012551	forward:5′cacctgaccacagagtcctttt3′ reverse: 5′aaagtgttgccactgttggg3′	152
*Eln*	NM_012722.1	forward: 5′gtgtcggtcttccaggtgta3′ reverse: 5′gaaccttggccttgactcct3′	117
*Fbn1*	NM_031825.1	forward: 5′gtgtgaactgagcgcgaac3′ reverse: 5‘cactggccaccatcacagata3′	288
*Fn1*	NM_019143.2	forward: 5′tcccacgatccgatgatgt3′ reverse: 5′tccacacggtatccagtcac3′	118
*Hmgb1*	NM_012963.2	forward: 5′aaggggagaccaaaaagaagtt3′ reverse: 5′acaagaagaaggccgaagga3′	69
*Il1a*	NM_017019.1	forward: 5′atccaacccagatcagcac3′ reverse: 5′actcctgcttgacgatcc3′	76
*Il1b*	NM_031512.2	forward: 5′cccattagacagctgcact3′ reverse: 5′ccattgaggtggagagctt3′	95
*Il33*	NM_001014166.1	forward: 5′gtggatgggaagaagctga3′ reverse: 5′tgaagaacaaagaaggcctga3′	140
*Il6*	M26744.1	forward: 5′tgccttcttgggactgatg3′ reverse: 5′tggtctgttgtgggtggt3′	97
*Mkx*	XM_017600733.1	forward: 5′gctctaggctcgcagatgac3′ reverse: 5′gcgttgccctgaacatactt3′	143
*Mmp1*	NM_001134530.1	forward: 5′gggtttttgaggaggaaggtg3′ reverse: 5′gcctggtgggaatgtgtga3′	113
*Mmp3*	NM_133523.3	forward: 5′cggtggcttcagtaccttt3′ reverse: 5′tcacctcctcccagacctt3′	143
*Mmp9* [81]	NM_031055.1	forward: 5′tgctcctggctctaggctac3′ reverse: 5′ttggaggttttcaggtctcg3′	88
*Ptger4*	NM_032076.3	forward: 5′gcgcaaggagcagaaggaga3′ reverse: 5′ccagcccacataccagagt3′	50
*Ptges2*	NM_001107832.1	forward: 5′aggacggaggagatgaagt3′ reverse: 5′cgttgggagagatgagatgc3′	67
*Postn*	NM_001108550	forward: 5′tagggtgtgagggagacagc3′ reverse: 5′caggtccgtgaaagtggttt3′	170
*Rps18*	NM_213557.1	forward: 5′tgtggtgttgaggaaagcag3′ reverse: 5′cctctatgggctcggatttt3′	240
*Scx*	NM_001130508.1	Qiagen (QT01596028), no primer sequence provided	
*Tnc*	NM_053861.1	forward: 5′atgttccaaagagccagcaa3′ reverse: 5′aggctgtagttgaggcggta3′	247
*Tac1*	NM_012666.2	forward: 5′caatgcagaactacgaaagaagg3′ reverse: 5′gcggacacagatggagatga3′	73
*Tac1r*	NM_012667.2	forward: 5′caaacgcaaggtggtcaaaa3′ reverse: 5′gatgtagggcaggaggaagaa3′	94
*Tnfa*	NM_012675.3	forward: 5′gcctcttctcattcctgct3′ reverse: 5′aacttctcctccttgttggg3′	97
*Tgfb1*	NM_021578.2	forward: 5′aactgtggagcaacacgtagaa3′ reverse: 5′tattccgtctccttggttcag3′	157
*Tgfb3*	NM_013174.2	forward: 5′gagggtggaagccattagg3′ reverse: 5′gcagactgccagttcattgtg3′	256
*Tnmd*	NM_022290.1	forward:5′ggcccgaggtatccaagaag3′ reverse: 5′agatgccagtgtatccgttttt3′	177

## Data Availability

The data sets of the present study are available from the corresponding author upon justified request.

## References

[1] Kujala U.M., Sarna S., Kaprio J. (2005). Cumulative Incidence of Achilles Tendon Rupture and Tendinopathy in Male Former Elite Athletes. Clin. J. Sport Med..

[2] Waldecker U., Hofmann G., Drewitz S. (2012). Epidemiologic investigation of 1394 feet: Coincidence of hindfoot malalignment and Achilles tendon disorders. Foot Ankle Surg..

[3] Riel H., Lindstrøm C.F., Rathleff M.S., Jensen M.B., Olesen J.L. (2019). Prevalence and incidence rate of lower-extremity tendinopathies in a Danish general practice: A registry-based study. BMC Musculoskelet. Disord..

[4] Maffulli N. (1998). Overuse tendon conditions: Time to change a confusing terminology. Arthrosc. J. Arthrosc. Relat. Surg..

[5] Scott A., Cook J.L., Hart D.A., Walker D.C., Duronio V., Khan K.M. (2007). Tenocyte responses to mechanical loading in vivo: A role for local insulin-like growth factor 1 signaling in early tendinosis in rats. Arthritis Rheum..

[6] Rees J.D., Maffulli N., Cook J. (2009). Management of Tendinopathy. Am. J. Sports Med..

[7] Maffulli N., Wong J., Almekinders L.C. (2003). Types and epidemiology of tendinopathy. Clin. Sports Med..

[8] Kaux J.-F., Forthomme B., Le Goff C., Crielaard J.-M., Croisier J.-L. (2011). Current Opinions on Tendinopathy. J. Sports Sci. Med..

[9] O’Neill S., Watson P.J., Barry S. (2021). A delphi study of risk factors for achilles tendinopathy- opinions of world tendon experts. Int. J. Sports Phys. Ther..

[10] Järvinen T.A., Kannus P., Maffulli N., Khan K.M. (2005). Achilles Tendon Disorders: Etiology and Epidemiology. Foot Ankle Clin..

[11] Abate M., Schiavone C., Salini V., Andia I. (2013). Occurrence of tendon pathologies in metabolic disorders. Rheumatol..

[12] Abate M., Gravare-Silbernagel K., Siljeholm C., Di Iorio A., De Amicis D., Salini V., Werner S., Paganelli R. (2009). Pathogenesis of tendinopathies: Inflammation or degeneration?. Arthritis Res. Ther..

[13] Fredberg U., Stengaard-Pedersen K. (2008). Chronic tendinopathy tissue pathology, pain mechanisms, and etiology with a special focus on inflammation. Scand. J. Med. Sci. Sports.

[14] D’Addona A., Maffulli N., Formisano S., Rosa D. (2017). Inflammation in tendinopathy. Surg..

[15] Rees J.D., Stride M., Scott A. (2014). Tendons–time to revisit inflammation. Br. J. Sports Med..

[16] Kannus P. (2000). Structure of the tendon connective tissue. Scand. J. Med. Sci. Sports.

[17] Gracey E., Burssens A., Cambré I., Schett G., Lories R., McInnes I.B., Asahara H., Elewaut D. (2020). Tendon and ligament mechanical loading in the pathogenesis of inflammatory arthritis. Nat. Rev. Rheumatol..

[18] Sugiyama Y., Naito K., Goto K., Kojima Y., Furuhata A., Igarashi M., Nagaoka I., Kaneko K. (2019). Effect of aging on the tendon structure and tendon-associated gene expression in mouse foot flexor tendon. Biomed. Rep..

[19] Lin D., Alberton P., Caceres M.D., Volkmer E., Schieker M., Docheva D. (2017). Tenomodulin is essential for prevention of adipocyte accumulation and fibrovascular scar formation during early tendon healing. Cell Death Dis..

[20] Klatte-Schulz F., Minkwitz S., Schmock A., Bormann N., Kurtoglu A., Tsitsilonis S., Manegold S., Wildemann B. (2018). Different Achilles Tendon Pathologies Show Distinct Histological and Molecular Characteristics. Int. J. Mol. Sci..

[21] Ireland D., Harrall R., Curry V., Holloway G., Hackney R., Hazleman B., Riley G. (2001). Multiple changes in gene expression in chronic human Achilles tendinopathy. Matrix Biol..

[22] Kostrominova T.Y., Brooks S.V. (2013). Age-related changes in structure and extracellular matrix protein expression levels in rat tendons. AGE.

[23] Magra M. (2005). Matrix metalloproteases: A role in overuse tendinopathies. Br. J. Sports Med..

[24] Fu S.C., Chan B.P., Wang W., Pau H.M., Chan K.M., Rolf C.G. (2002). Increased expression of matrix metalloproteinase 1 (MMP1) in 11 patients with patellar tendinosis. Acta Orthop. Scand..

[25] Alfredson H., Lorentzon M., Bäckman S., Bäckman A., Lerner U. (2003). cDNA-arrays and real-time quantitative PCR techniques in the investigation of chronic achilles tendinosis. J. Orthop. Res..

[26] Thampatty B.P., Li H., Im H.-J., Wang J.H.-C. (2007). EP4 receptor regulates collagen type-I, MMP-1, and MMP-3 gene expression in human tendon fibroblasts in response to IL-1β treatment. Gene.

[27] Tsuzaki M., Guyton G., Garrett W., Archambault J.M., Herzog W., Almekinders L., Bynum D., Yang X., Banes A.J. (2003). IL-1β induces COX2, MMP-1, -3 and -13, ADAMTS-4, IL-1β and IL-6 in human tendon cells. J. Orthop. Res..

[28] Bergqvist F., Carr A.J., Wheway K., Watkins B., Oppermann U., Jakobsson P.-J., Dakin S.G. (2019). Divergent roles of prostacyclin and PGE2 in human tendinopathy. Arthritis Res..

[29] Dakin S., Dudhia J., Werling N., Werling D., Abayasekara D.R.E., Smith R.K.W. (2012). Inflamm-Aging and Arachadonic Acid Metabolite Differences with Stage of Tendon Disease. PLoS ONE.

[30] Sullo A., Maffulli N., Capasso G., Testa V. (2001). The effects of prolonged peritendinous administration of PGE1 to the rat Achilles tendon: A possible animal model of chronic Achilles tendinopathy. J. Orthop. Sci..

[31] Khan M.H., Li Z., Wang J.H.-C. (2005). Repeated Exposure of Tendon to Prostaglandin-E2 Leads to Localized Tendon Degeneration. Clin. J. Sport Med..

[32] Movin T., Gad A., Reinholt F.P., Rolf C. (1997). Tendon pathology in long-standing achillodynia: Biopsy findings in 40 patients. Acta Orthop. Scand..

[33] Koch L.G., Britton S.L. (2001). Artificial selection for intrinsic aerobic endurance running capacity in rats. Physiol. Genom..

[34] Noland R.C., Thyfault J., Henes S.T., Whitfield B.R., Woodlief T., Evans J.R., Lust J.A., Britton S.L., Koch L.G., Dudek R.W. (2007). Artificial selection for high-capacity endurance running is protective against high-fat diet-induced insulin resistance. Am. J. Physiol. Metab..

[35] Wisløff U., Najjar S.M., Ellingsen Ø., Haram P.M., Swoap S., Al-Share Q., Fernstro M., Rezaei K., Lee S.J., Koch L.G. (2005). Cardiovascular Risk Factors Emerge After Artificial Selection for Low Aerobic Capacity. Science.

[36] Schwarzer M., Britton S.L., Koch L.G., Wisloff U., Doenst T. (2010). Low intrinsic aerobic exercise capacity and systemic insulin resistance are not associated with changes in myocardial substrate oxidation or insulin sensitivity. Basic Res. Cardiol..

[37] Koch L.G., Kemi O.J., Qi N., Leng S.X., Bijma P., Gilligan L.J., Wilkinson J.E., Wisløff H., Høydal M.A., Rolim N. (2011). Intrinsic Aerobic Capacity Sets a Divide for Aging and Longevity. Circ. Res..

[38] Longo U.G., Berton A., Stelitano G., Madaudo C., Perna M., Ciuffreda M., Guarnieri A., Papalia R., Maffulli N., Denaro V. (2018). 2017 Marathon of Rome. Clin. J. Sport Med..

[39] Zhou B., Zhou Y., Tang K. (2014). An overview of structure, mechanical properties, and treatment for age-related tendinopathy. J. Nutr. Heal. Aging.

[40] Abate M., Salini V. (2019). Mid-portion Achilles tendinopathy in runners with metabolic disorders. Eur. J. Orthop. Surg. Traumatol..

[41] Speed C. (2016). Inflammation in Tendon Disorders. Advances in Experimental Medicine and Biology.

[42] Yu T.-Y., Pang J.-H.S., Wu K.P.-H., Chen M.J.-L., Chen C.-H., Tsai W.-C. (2013). Aging is associated with increased activities of matrix metalloproteinase-2 and -9 in tenocytes. BMC Musculoskelet. Disord..

[43] Pingel J., Fredberg U., Qvortrup K., Larsen J.O., Schjerling P., Heinemeier K., Kjaer M., Langberg H. (2012). Local biochemical and morphological differences in human Achilles tendinopathy: A case control study. BMC Musculoskelet. Disord..

[44] Corps A.N., Robinson A.H.N., Movin T., Costa M.L., Hazleman B.L., Riley G.P. (2006). Increased expression of aggrecan and biglycan mRNA in Achilles tendinopathy. Rheumatol..

[45] Minkwitz S., Schmock A., Kurtoglu A., Tsitsilonis S., Manegold S., Wildemann B., Klatte-Schulz F. (2017). Time-Dependent Alterations of MMPs, TIMPs and Tendon Structure in Human Achilles Tendons after Acute Rupture. Int. J. Mol. Sci..

[46] Riley G.P., Curry V., DeGroot J., van El B., Verzijl N., Hazleman B.L., Bank R.A. (2002). Matrix metalloproteinase activities and their relationship with collagen remodelling in tendon pathology. Matrix Biol..

[47] Koskinen S.O.A., Heinemeier K.M., Olesen J.L., Langberg H., Kjaer M. (2004). Physical exercise can influence local levels of matrix metalloproteinases and their inhibitors in tendon-related connective tissue. J. Appl. Physiol..

[48] Koskinen S.O.A., Hoyhtya M., Turpeenniemi-Hujanen T., Martikkala V., Makinen T.T., Oksa J., Rintamaki H., Lofberg M., Somer H., Takala T.E.S. (2001). Serum concentrations of collagen degrading enzymes and their inhibitors after downhill running. Scand. J. Med. Sci. Sports.

[49] Alberton P., Dex S., Popov C., Shukunami C., Schieker M., Docheva D. (2015). Loss of Tenomodulin Results in Reduced Self-Renewal and Augmented Senescence of Tendon Stem/Progenitor Cells. Stem Cells Dev..

[50] Dex S., Alberton P., Willkomm L., Söllradl T., Bago S., Milz S., Shakibaei M., Ignatius A., Bloch W., Clausen-Schaumann H. (2017). Tenomodulin is Required for Tendon Endurance Running and Collagen I Fibril Adaptation to Mechanical Load. EBioMedicine.

[51] Guerquin M.-J., Charvet B., Nourissat G., Havis E., Ronsin O., Bonnin M.-A., Ruggiu M., Olivera-Martinez I., Robert N., Lu Y. (2013). Transcription factor EGR1 directs tendon differentiation and promotes tendon repair. J. Clin. Investig..

[52] Abraham A.C., Shah S.A., Golman M., Song L., Li X., Kurtaliaj I., Akbar M., Millar N.L., Abu-Amer Y., Galatz L.M. (2019). Targeting the NF-κB signaling pathway in chronic tendon disease. Sci. Transl. Med..

[53] Buhrmann C., Mobasheri A., Busch F., Aldinger C., Stahlmann R., Montaseri A., Shakibaei M. (2011). Curcumin Modulates Nuclear Factor κB (NF-κB)-mediated Inflammation in Human Tenocytes in Vitro. J. Biol. Chem..

[54] Liu T., Zhang L., Joo D., Sun S.-C. (2017). NF-κB signaling in inflammation. Signal Transduct. Target. Ther..

[55] Fu S.C., Wang W., Pau H.M., Wong Y.P., Chan K.M., Rolf C.G. (2002). Increased Expression of Transforming Growth Factor-??1 in Patellar Tendinosis. Clin. Orthop. Relat. Res..

[56] Legerlotz K., Jones E.R., Screen H., Riley G. (2012). Increased expression of IL-6 family members in tendon pathology. Rheumatol..

[57] Murakami M., Nakatani Y., Tanioka T., Kudo I. (2002). Prostaglandin E synthase. Prostaglandins Other Lipid Mediat..

[58] Zhang J., Wang J.H.-C. (2009). Production of PGE2increases in tendons subjected to repetitive mechanical loading and induces differentiation of tendon stem cells into non-tenocytes. J. Orthop. Res..

[59] Han S.-H., Choi W., Song J., Kim J., Lee S., Choi Y., Byun S.-E., Ahn T., Ahn H., Ding C. (2017). The Implication of Substance P in the Development of Tendinopathy: A Case Control Study. Int. J. Mol. Sci..

[60] Andersson G., Backman L.J., Scott A., Lorentzon R., Forsgren S., Danielson P. (2011). Substance P accelerates hypercellularity and angiogenesis in tendon tissue and enhances paratendinitis in response to Achilles tendon overuse in a tendinopathy model. Br. J. Sports Med..

[61] Backman L.J., Fong G., Andersson G., Scott A., Danielson P. (2011). Substance P Is a Mechanoresponsive, Autocrine Regulator of Human Tenocyte Proliferation. PLoS ONE.

[62] Oh S.Y., Kim D.K., Han S.H., Lee H.H., Jeong Y., Baek M., Kim H., Ahn W., Lee S. (2020). Sustained Exposure of Substance P Causes Tendinopathy. Int. J. Mol. Sci..

[63] Scott A. (2009). Neuropeptides in tendinopathy. Front. Biosci..

[64] Andersson G., Danielson P., Alfredson H., Forsgren S. (2008). Presence of substance P and the neurokinin-1 receptor in tenocytes of the human Achilles tendon. Regul. Pept..

[65] Molloy T., Wang Y., Murrell G.A.C. (2003). The Roles of Growth Factors in Tendon and Ligament Healing. Sports Med..

[66] Tran P.H.T., Malmgaard-Clausen N.M., Puggaard R.S., Svensson R.B., Nybing J.D., Hansen P., Schjerling P., Zinglersen A.H., Couppé C., Boesen M. (2020). Early development of tendinopathy in humans: Sequence of pathological changes in structure and tissue turnover signaling. FASEB J..

[67] Goodier H.C.J., Carr A.J., Snelling S.J.B., Roche L., Wheway K., Watkins B., Dakin S.G. (2016). Comparison of transforming growth factor beta expression in healthy and diseased human tendon. Arthritis Res..

[68] Meller R., Schiborra F., Brandes G., Knobloch K., Tschernig T., Hankemeier S., Haasper C., Schmiedl A., Jagodzinski M., Krettek C. (2009). Postnatal maturation of tendon, cruciate ligament, meniscus and articular cartilage: A histological study in sheep. Ann. Anat.-Anat. Anz..

[69] Almekinders L.C., Deol G. (1999). The Effects of Aging, Antiinflammatory Drugs, and Ultrasound on the In Vitro Response of Tendon Tissue. Am. J. Sports Med..

[70] Svensson R.B., Heinemeier K.M., Couppé C., Kjaer M., Magnusson S.P. (2016). Effect of aging and exercise on the tendon. J. Appl. Physiol..

[71] Bell R., Gendron N.R., Anderson M., Flatow E.L., Andarawis-Puri N. (2018). A potential new role for myofibroblasts in remodeling of sub-rupture fatigue tendon injuries by exercise. Sci. Rep..

[72] Junqueira L.C.U., Cossermelli W., Brentani R. (1978). Differential Staining of Collagens Type I, II and III by Sirius Red and Polarization Microscopy. Arch. Histol. Jpn..

[73] Junqueira L.C.U., Bignolas G., Brentani R.R. (1979). Picrosirius staining plus polarization microscopy, a specific method for collagen detection in tissue sections. Histochem. J..

[74] Fleischhacker V., Klatte-Schulz F., Minkwitz S., Schmock A., Rummler M., Seliger A., Willie B.M., Wildemann B. (2020). In Vivo and In Vitro Mechanical Loading of Mouse Achilles Tendons and Tenocytes—A Pilot Study. Int. J. Mol. Sci..

[75] Montes G.S., Junqueira L.C.U. (1991). The use of the Picrosirius-polarization method for the study of the biopathology of collagen. Memórias do Instituto Oswaldo Cruz.

[76] Lattouf R., Younes R., Lutomski D., Naaman N., Godeau G., Senni K., Changotade S. (2014). Picrosirius Red Staining. J. Histochem. Cytochem..

[77] Dirks R.C., Galley M.R., Childress P.J., Fearon A.M., Scott A., Koch L.G., Britton S.L., Warden S.J. (2013). Uphill running does not exacerbate collagenase-induced pathological changes in the Achilles tendon of rats selectively bred for high-capacity running. Connect. Tissue Res..

[78] Kohn D.F., Clifford C.B. (2002). Biology and Diseases of Rats.

[79] Quinn R. (2005). Comparing rat’s to human’s age: How old is my rat in people years?. Nutrition.

[80] Russell W.M.S., Burch R.L. (1959). The Principles of Humane Experimental Technique.

[81] Deschner J., Rath-Deschner B., Agarwal S. (2006). Regulation of matrix metalloproteinase expression by dynamic tensile strain in rat fibrochondrocytes. Osteoarthr. Cartil..

[82] Simon P. (2003). Q-Gene: Processing quantitative real-time RT-PCR data. Bioinformation.

[83] Bustin S.A., Benes V., Garson J.A., Hellemans J., Huggett J., Kubista M., Mueller R., Nolan T., Pfaffl M.W., Shipley G.L. (2009). The MIQE Guidelines: Minimum Information for Publication of Quantitative Real-Time PCR Experiments. Clin. Chem..

